# Application of Single-Cell Sequencing Technology in Research on Colorectal Cancer

**DOI:** 10.3390/jpm14010108

**Published:** 2024-01-18

**Authors:** Long Zhao, Quan Wang, Changjiang Yang, Yingjiang Ye, Zhanlong Shen

**Affiliations:** 1 Department of Gastroenterological Surgery, Peking University People’s Hospital, Beijing 100044, China; 2111110407@pku.edu.cn (L.Z.); 2211110425@stu.pku.edu.cn (C.Y.); yeyingjiang@pkuph.edu.cn (Y.Y.); 2 Laboratory of Surgical Oncology, Peking University People’s Hospital, Beijing 100044, China; 3 Department of Ambulatory Surgery Center, Xijing Hospital, Air Force Military Medical University, Xi’an 710032, China; wangquan2013@fmmu.edu.cn

**Keywords:** colorectal cancer, single-cell sequencing, heterogeneity, immune microenvironment, therapy

## Abstract

Colorectal cancer (CRC) is the third most prevalent and second most lethal cancer globally, with gene mutations and tumor metastasis contributing to its poor prognosis. Single-cell sequencing technology enables high-throughput analysis of the genome, transcriptome, and epigenetic landscapes at the single-cell level. It offers significant insights into analyzing the tumor immune microenvironment, detecting tumor heterogeneity, exploring metastasis mechanisms, and monitoring circulating tumor cells (CTCs). This article provides a brief overview of the technical procedure and data processing involved in single-cell sequencing. It also reviews the current applications of single-cell sequencing in CRC research, aiming to enhance the understanding of intratumoral heterogeneity, CRC development, CTCs, and novel drug targets. By exploring the diverse molecular and clinicopathological characteristics of tumor heterogeneity using single-cell sequencing, valuable insights can be gained into early diagnosis, therapy, and prognosis of CRC. Thus, this review serves as a valuable resource for identifying prognostic markers, discovering new therapeutic targets, and advancing personalized therapy in CRC.

## 1. Introduction

Since Hooke discovered cells in 1665, cells have been studied for more than 150 years, but numerous cell subtypes and states remain unknown [1]. To obtain high-resolution information on cell type, quantity, location, relationship, and molecular expression for accurately defining cell composition and the status of health or disease, a project (human cell atlas, HCA) with epochal significance was proposed [2]. However, traditional bulk sequencing methods only offer comparisons between two types of tissues between different sources and fail to study the heterogeneity between cells in tissues. Moreover, the nature of gene expression is not adequately researched. This is because in traditional sequencing, tissue samples contain thousands of cells, which eventually generalizes the gene signals from all cell subpopulations in this group or just reveals the genetic information of the predominant cells, while ignoring the unique genetic information of a single cell [3]. To overcome the shortcomings of traditional sequencing, single-cell transcriptomic sequencing (SCS) technology based on an omnidirectional, multi-level, and high-throughput technique was developed. SCS can be used for examination of the gene sequence and transcript, protein, and epigenetic expression profiles at a single-cell level. Through functional analysis, genetic and expression information can be linked with cell behavior. Using spatial mapping, cells can be located within certain tissues or organs, and a map of different types of cells can be obtained [4,5]. The discovery of SCS not only made the measurement of gene expression more accurate, but also facilitated the detection of rare non-coding RNA expression, even at low expression levels. It is also useful for sequencing special samples, such as tumor circulating cells, tissue microarrays, early developing embryonic cells, and samples that do not meet the requirements of whole-genome sequencing [6,7]. It has been successfully used in many fields such as tumor typing, targeted drug use, immunotherapy, embryonic development in animal and plant, and cardiovascular diseases research [6,8,9,10,11].

Colorectal cancer (CRC) has a high tumor mutational burden (TBM), and ranks the second in tumor-related mortality [12]. The latest data predict that approximately 148,000 people are diagnosed with CRC annually in the United States, and the predicted number of deaths is approximately 43,000 [13]. Tumor heterogeneity refers to the difference in genetic and molecular characteristics among cancer cells owing to the different degrees of cellular differentiation [14]. As a complex whole, tumor heterogeneity widely exists in tumors and plays an important role in the genesis and development of cancers [15,16], which increases the challenges in targeted therapy [17].

CRC is highly heterogeneous [18,19]. Using traditional bulk transcriptomics to measure total gene expression in heterogeneous tissues fails to reveal the complexity between the interaction of the transcriptome and proportions of different cells within the tumor microenvironment as well as the variation within tumors at the single-cell level [20]. Conversely, in single-cell isolation and sequencing, a single cell is used as the research object [21]. The heterogeneity of CRC cells can be investigated more thoroughly using this method. Single-cell separation technology combined with high-throughput sequencing facilitates the analysis of the diverse molecular characteristics of tumor tissues and can be applied not only for basic research, but also in clinical diagnosis and drug development. In addition, it can also be used to identify abnormal cell proliferation to identify novel pathogenetic mechanisms in CRC [22].

## 2. Overview of Single-Cell Sequencing Technology

### 2.1. Sample Preparation and Single Cell Isolation

Compared to traditional sequencing samples, SCS samples have stricter requirements. In the common SCS sample preparation method, cells separated from fresh tissue samples are favored to avoid the impacts of ischemia and hypoxia on the tissue and cell status after isolation. Concurrently, the simultaneous processing after the collection of multiple samples at different time points can help minimize the technical batch effect [23].

Single-cell separation techniques can be classified into various categories. After single-cell separation and dissolution, pg-grade nucleic acids can be obtained and subsequently amplified to ng or μg grade for subsequent sequencing. Various single-cell separation methods have been developed based on the state of the sample, number of cells needed, and purpose of analysis, such as laser capture microdissection (LCM) [24], fluorescence activating cell sorter (FACS) [25], magnetic-activated cell sorting (MACS) [26], and others.

Sample preparation and single cell isolation for single-cell sequencing involve several crucial steps. Initially, solid tissues are dissociated into single-cell suspension to release intact and viable cells while minimizing damage. The resulting suspension is filtered and washed to remove cell clumps and debris, followed by optional cell sorting methods to isolate specific cell populations. Subsequently, single cells are captured using microfluidic systems or alternative approaches with quality control ensuring the presence of single, viable cells without contamination. Reverse transcription and pre-amplification are performed on isolated cells to convert RNA into cDNA and generate sufficient material for subsequent steps. During library preparation, adapters are added to the cDNA fragments, facilitating sequencing. The prepared libraries are subjected to high-throughput sequencing, generating large amounts of sequencing data. Bioinformatics analysis is then conducted to align reads, quantify expression, detect genetic variants, and explore molecular features at the single-cell level. The specific techniques utilized may vary based on experimental requirements, cell types, and sequencing technologies.

### 2.2. Summary of Single-Cell Sequencing Technology

Single-cell genome sequencing uses an accurate separation technology to isolate individual cells, extract and amplify DNA using new-generation whole-genome amplification (WGA) technology. This process allows to generate the whole-genome map of single cells, which is primarily used to reveal dissimilarities among cell subsets and their relationship to cell evolution. SCS technology was first reported by Surani and Tang in 2009 [27]. However, it was not until 2014 that this method gained widespread recognition due to the improvement of this method and a significant decrease in sequencing cost. Table 1 summarizes the SCS techniques currently in use.

Since Tang et al. first utilized single-cell sequencing technology to analyze the cDNA expression profile of single mouse embryonic cells in 2009, single-cell sequencing has continuously undergone technological innovations, and a series of new sequencing technologies have emerged, such as Smart-seq [33], 10× Genomics [34], and Quartz-Seq [30]. Single-cell separation is one of the most significant steps in the evolution of single-cell sequencing technology. Traditional methods for cell isolation include serial dilution, fluorescence-activated cell sorting (FACS), and laser capture microdissection (LCM). However, with the creation of high-throughput sequencing platforms, cell sorting platforms based on magnetically-activated cell sorting (MACS) and microfluidics have been developed, which significantly improved the efficiency and precision of single-cell isolation [35]. Due to the ability of scRNA-Seq to measure the entire transcriptome at the resolution of single cells and capture the transcriptional characteristics of single cells, it has been widely utilized in tumor research, including colorectal cancer. The evolution of single-cell sequencing technology has enabled researchers to explore the development of different tumor cells, construct a blueprint of the tumor cell microenvironment, discover new biomarkers and therapeutic targets, and elucidate the underlying mechanisms, which could facilitate the development of combination therapy strategies for cancer patients [22].

However, there are still some limitations within the current single-cell sequencing technology. Firstly, SCS requires a relatively high level of sample numbers and quality, including adequate cell quantity and activity, which could cause an unaffordable overall cost. In addition, although the number of cells detectable by SCS has significantly increased along with technological advancements, current sample preparation processes can still result in the loss of certain cell populations, which potentially could bring bias into the results [36].

Epigenetic information refers to the heritable genetic information beyond the DNA sequence, including DNA methylation, RNA methylation, histone modification, chromatin remodeling, and three-dimensional conformation data, among which DNA methylation analysis is a relatively commonly employed epigenetic method. Single-cell reduced-representation bisulfite sequencing technology can be used to identify CpG islands using the restriction enzyme MspI to identify CpG islands and enrich fragments of genes at specific sites. This method can be used for detecting single-base covering CpG islands at the single-cell level. Research on single-cell proteomics can be traced to 2004, and the method was proposed by Nolan and Dovichi [37,38]. However, the technical focus was different. Nolan used flow cytometry to capture cell signals at the single-cell level, while Dovichi used chemical cytology to obtain a single cell by capillary electrophoresis based on fluorescence labeling. Later, it developed into a single-cell protein sequencing method. In addition, the current method of single-cell multi-omics joint analysis can be used to obtain genome, epigenome, transcriptome, and proteome information from the same cell, which can help integrate the understanding of multi-omics data on a single cell [39].

### 2.3. Applications of Single-Cell Sequencing in Multi-Omics Analysis

Since single-cell sequencing could provide the crucial information of single-cell transcriptome, genome, and epigenome, which creates a platform for multi-omics analysis, especially for the comprehensive study of cancers. One robust example is called TARGET-seq, which utilizes flow cytometry to sort cells into microplates and extracts gDNA and mRNA during cell lysis, then the specific primers for gDNA and cDNA, respectively are used for scDNA-seq and scRNA-seq. Sun et al. [40] used TARGET-seq to analyze the transcriptional and genetic heterogeneity of the myeloproliferative neoplasms (MPNs) and found the novel mechanisms of both mutated and nonmutated cell dysregulation in tumor progression. Another combination is the analysis of the transcriptome with epigenome. Single-cell triple omics sequencing (scTrio-seq) could investigate cellular CNV, transcriptome and DNA methylome at the same time [41]. The key process of scTrio-seq is the mild lysis protocol that can retain the nucleus DNA as well as cytoplasmic mRNA, and through centrifugation, the cytoplasmic mRNA is subjected to scRNA-seq, while the precipitate containing gDNA is subjected to single-cell reduced representation bisulfite sequencing (scRRBS) for DNA methylome analysis [42].

### 2.4. Identification and Analysis of Cell Subsets

After SCS results are obtained, the specific expression of genes or proteins in specific cell subsets after specific grouping can be identified. The determination of the specific grouping of cell subsets is crucial, and typically involves manual categorization. First, it is necessary to determine the cell type and corresponding markers in the sample. One can refer to existing literature or databases to confirm the cell type in the sample tissue. Compared with traditional sequencing, which only identifies a few marker genes in cells, SCS can help accurately identify a large number of marker genes in cells.

After the identification of cell subpopulations, differential analysis of gene expression can be conducted for specific cell subpopulations. This method is consistent with the second-generation sequencing algorithm. Because SCS can be further refined to the single-cell level, quasi-time series analysis methods can be applied to study the trajectory of cell differentiation. With the discovery of new cell types in single cells, we can identify the sub-subtypes of known cell subsets and intermediate transition cell sub-subsets based on the trajectory of cell evolution. Additionally, this could help identify rare cell types that have not been defined, which are relatively difficult to define and verify in the current SCS field. At present, the published research contents of CSC primarily focus on cell heterogeneity in tissues, gene screening in specific cell subsets, and validation of downstream targets.

## 3. Single-Cell Sequencing in Colorectal Cancer

### 3.1. Colorectal Cancer Cell Heterogeneity

CRC is a highly heterogeneous disease, with intratumoral heterogeneity a major obstacle to effective treatment and accurate tumor analysis [43]. Dai et al. [44] performed scRNA-seq analysis on tumor tissues of patients with CRC. Their analysis of 2824 CRC cells showed five different subgroups, and each subgroup could correspond to specific cell markers, indicating that there was significant heterogeneity among different subgroups of CRC tumor epithelial cells. Concurrently, Chen et al. [43] proposed that single-cell analysis not only summarizes the findings of single-nucleotide polymorphism analysis in large samples, but also helps detect variation between cells, which is helpful for early diagnosis and discovering potential mechanisms underlying cancer development at the single-cell level. Adalsteinsson et al. [45] used microporous array technology to investigate the secretion of alpha chemokines (ELR + CXC) in single colorectal epithelial tumor cells and stromal cells according to the presence of the glutamate-arginine-leucine (ELR) sequence to characterize the secretion rate of various factors and the number of cells secreting each factor. The secretory state among human primary colorectal tumors, stromal cells, and cell lines was complex and could evolve dynamically. Their findings provided novel insights for assessing the intratumoral phenotypic heterogeneity in human primary tumors. Concurrently, Li et al. [43] conducted SCS on 11 primary colorectal tumors and their matched normal mucosa and identified two different subsets of cancer-associated fibroblasts (CAFs). The findings showed that epithelial-mesenchymal transformation (EMT)-related genes were only upregulated in the certain CAF subsets of tumors. In addition, considering CRC’s heterogeneous molecular subtypes, tumor cells even within the same tumor could also exhibit intratumoral heterogeneity.

Liu et al. [46] sequenced the whole genome and performed single-cell genome sequencing across multiple regions of the same tumor. By sequencing nine tumor regions and 88 single cells of two patients with rectal cancer according to the same molecular classification, they characterized mutation spectrum and changes in the somatic cell copy number at multiple regions and single-cell levels, revealing that each tumor had its own internal microenvironment, which may lead to different diagnoses. This could be the primary cause of prognosis and drug reaction. Zhang et al. [47] analyzed the SCS results of 272 CRC epithelial cells and 160 normal epithelial cells and identified 342 differentially expressed transcripts using machine learning methods. The 342 transcripts were further divided into two categories. Transcript upregulation in CRC epithelial cells was significantly enriched in the ribosome pathway, protein processing, antigen processing, and presentation in endoplasmic reticulum, p53, and other signaling pathways.

Recently, the Iain Beehuat Tan research team at the Singapore Genome Research Institute reported two new classifications for CRC, iCMS2 and iCMS3, based on epithelial cell characteristics through the single-cell transcriptome analysis of CRC samples from patients [48]. Based on these insights, the authors proposed a new refined classification of CRC, offering a new tool for developing patient treatment plans and conducting prognosis analysis.

### 3.2. Colorectal Cancer Genetic Evolution

SCS is a powerful technique to decipher clonal evolutionary relationships and identify key driving genes. Yu et al. [49] conducted CSC analysis on colon cancer and determined two independent clonal subsets within the tumor cell population. Among them, the major tumor clone subgroups demonstrated early carcinogenic factors, such as APC and TP53 gene mutations, whereas the minor clone exhibited major CDC27 and PABPC1 mutations. The absence of APC and TP53 mutations in the secondary clone indicated that these two clones originated from two distinct cell origins. Concurrently, the somatic mutation allele spectrum analysis across 21 other colon cancer tissues revealed the heterogeneity of clonal origin of colon cancers. Unexpectedly, SLC12A5 was found to have a higher mutation frequency at the single-cell level but a lower mutation rate at the population level. The function of mutant SLC12A5 showed a potential carcinogenic role in colon cancer. At the same time, Liu et al. [46] studied CRC-related tumor stem cells, applied fluorescence-activated cell sorting technology, and separated two CSC subsets that expressed epithelial cell adhesion molecules (EpCAMhi) and extracellular matrix receptor III (CD44+) at high levels from the tumor tissues of two patients with primary colon cancer. However, the subsets did not express protein tyrosine phosphatase receptor C (CD45−), and only expressed EpCAMhi at high levels. Somatic copy number changes (SCNA) in SCS and differentiated tumor cells (DTCs) were compared at the single-cell level. The main SCNA occurred in the early stage and was stably inherited. The SCNA similarity between SCS and DTCs may be attributed to lineage differentiation. Moreover, SCS from the same patient showed a reproducible SCNA map, indicating that the development of colon cancer involved some gene mutations.

Patient-derived organoid culture is a powerful tool to study the molecular mechanism under CRC. Fu et al. [50] established CRC organoids from patients and sequenced single-cell RNA from the organoids of seven patients, and the corresponding tumors and normal tissues. They found that the conditional medium was superior to the chemical and long-term media in maintaining the genomic, epigenomic, and transcriptomic characteristics of cancer cells as well as normal epithelial cells. The researchers also found that the cultured tumor-derived organoids could reflect the gene expression pattern, gene regulatory network, point mutation, CNV, and DNA methylation patterns of tumor cells in vitro. Conversely, normal tissue-derived organoids showed the normal genomic characteristics of normal epithelial cells in vitro but showed some tumor-like characteristics at the transcriptome level. The research group then used SCS and whole-exon sequencing to systematically analyze the dynamic changes in different cell types and the tumor subclonal composition, gene expression, signal pathway, genome copy number variation, and somatic mutation during the development and metastasis of CRC [51]. SCS results showed an overexpression of CEACAM6 and other genes, downregulation of CA2 and other genes, and significant activation of the PPAR signal pathway. SOX9+ MKI67+ double-positive cancer cells showed tumor stem cell-like biological characteristics. At the same time, it was confirmed at the single-cell level that tumor cells at different metastatic sites (lymph nodes and liver metastases, among others) in the same patient could have independent origin characteristics. The findings of this study provide a theoretical basis for understanding the malignant oncological behavior of colorectal cancer and develop the targeted drugs.

The tumor heterogeneity of CRC initiating cells (CRCICs) usually represents the invasive characteristics of tumor progression. For the high-resolution detection of CRCICs, Wu et al. [52] conducted single-cell whole exome sequencing and batch cell target exome sequencing for CRCICs to study stem cell-specific somatic changes or clonal evolution. The results showed that AHNAK2, PLIN4, HLA-B, ALK, CCDC92, and ALMS1 genes had specific mutations in CRCICs. In addition, four new predicted antigens of AHNAK2 were identified and verified, which provided practical value for the immunotherapy of patients with CRC.

### 3.3. Circulating Tumor Cells of Colorectal Cancer

Circulating tumor cells (CTCs) are highly dynamic and have a high potential for metastasis. They originate from the primary or metastatic lesions of epithelial-originated tumors and enter the bloodstream. Epithelial-mesenchymal transition (EMT) is an important mechanism in the progression of colorectal cancer (CRC), and CTCs are considered a powerful tool for revealing the mesenchymal phenotype transition potential and EMT-specific markers [53]. In addition, CTCs play a crucial role in liquid biopsy, providing a potential means for the real-time monitoring of tumor progression [54,55]. CTCs can also be used for exploring drug targets. For example, Smit et al. [56] established a stable CTC cell line, CTC-MCC-41, from CRC patients and found that low concentrations of Akt inhibitor MK2206 and mTOR inhibitor RAD001 could inhibit the growth of CTC-MCC-41, indicating that the PI3K/Akt/mTOR pathway may be a valuable drug target for inhibiting CRC metastasis. This evidence implied that the isolation and understanding of CTCs is helpful in exploring tumor progression, metastasis, and novel anti-tumor targets.

Single-cell sequencing is a critical technique for the detection of CTCs. Early applications using EpCAM as a capture platform for CTCs showed that CRC patients with higher expression of the EMT-related gene Akt-2 had a shorter median survival period. ALDH1, PI3Kα, and Akt-2 are independent variables for predicting disease-free survival in patients with metastatic CRC (mCRC) [57]. Li et al. [58] developed a novel non-specific adsorption immune magnetic platform called Fe3O4@SiO2@PTMAO@Aptamer, which efficiently differentiated CTC phenotypes during the EMT process, including epithelial-type CTCs, mesenchymal-type CTCs, and hybrid-type CTCs, in just 10 min. CTCs exhibit different phenotypes, including epithelial CTCs (E-CTCs), mesenchymal CTCs (M-CTCs), and mixed (epithelial and mesenchymal) CTCs (EM-CTCs). Using this platform, a study on 40 CRC patients found a strong correlation between the total number of CTCs, particularly M-CTCs, and tumor stage. The total number of CTCs, especially M-CTCs, served as a sensitive indicator for evaluating chemotherapy effectiveness. After tumor burden removal, a significant decrease in EM-CTC data was observed. This demonstrates the dynamic detection of EMT in CRC through CTCs, highlighting their close association with tumor progression, chemotherapy, and surgical outcomes. CTCs can serve as important tools for exploring intratumoral heterogeneity in CRC. Raimond et al. [59] reported heterogeneity in KRAS gene mutations between CRC tissues and CTCs, with a higher frequency of KRAS gene mutations in CTCs than in CRC tissues, and a lack of heterogeneity in KRAS gene mutation expression among paired samples. However, Bium et al. [60] obtained opposite results in their analysis of KRAS mutation characteristics in 26 mCRC patients, finding a certain consistency between the detection rates of KRAS mutations in CTCs and paired CRC tissues, independent of the clinical and pathological characteristics of CRC patients. It is worth noting that the sample sizes in the above studies were small, and larger sample studies are needed to confirm the existence of heterogeneity between CTCs and the primary tumor.

Above all, the single-cell sequencing of CTCs enables the simultaneous analysis of genomic, transcriptomic, and epigenetic differences between peripheral blood CTCs, primary tumors, and metastatic lesions, providing a new perspective for understanding the biological processes of CRC occurrence and development, as well as a new approach for identifying drug targets in CRC.

### 3.4. The Immune Microenvironment of Colorectal Cancer

The immune cells in the CRC tumor microenvironment (TME) can regulate tumor progression and are promising therapeutic targets [14]. As a key part of TME, T cells play a crucial role in cancer immunotherapy [61]. Zhang et al. [62] conducted comprehensive analysis of T cells in CRC samples using SCS to explore their characteristics and dynamic evolution. The researchers first isolated 11,138 T cells from 12 patients with CRC for the SCS and developed single T cell analysis by T cell receptor (TCR) tracking index to quantitatively analyze the dynamic relationship among 20 T cell subsets with different functions and clones. Simultaneously, to clarify the basic characteristics of tumor-infiltrating T cells, such as the functional status, migration capacity, and clone amplification, the researchers used Smart-seq2 to obtain 10,805 T cell expression profiles and 9878 cell TCR sequences from the peripheral blood, adjacent normal tissues, and the tumor tissues of 12 CRC patients. Using web-based application network tools (http://crctcell.cancer-pku.cn/, accessed on 11 January 2023), researchers characterized the TIL at the single-cell level based on transcriptome and assembled TCR sequences [63]. Conversely, in the TME, besides lymphocytes, myeloid immune cells, as the primary antigen-presenting cells, also play an important role in tumor immunity. However, the characteristics of these cells in the CRC TME and their interactions with T cells and other cells have not been fully explained. A research team examined the microenvironment landscape of tumor-infiltrating myeloid cells in patients with CRC at the single-cell level and analyzed the characteristics of the TAM and DC cell subsets, lineage development, and their interactions with T cells and other cells. Researchers found, for the first time, that the secreted phosphoprotein gene (SPP1++) TAM was specifically enriched in the cancer tissues of CRC patients, and revealed a similarity between anti-colony stimulating factor 1 receptor (Anti-CSF1R) treatment-tolerant TAM cell subsets and the SPP1++ TAM in patients with CRC, both showing overexpression of VEGFA, CD274, and Arg1 gene that involved in the immunosuppression process. Concurrently, the anti-CSF1R-blocking antibody could affect the proliferation of macrophages and specifically acted on macrophages with C1qC-chain related genes (C1QC++), but not on macrophages with SPP1+. This suggests that anti-CSF1R treatment retains macrophages that can promote tumor angiogenesis in tumors after treatment [64]. The findings could provide a mechanistic explanation for the limited efficacy of anti-CSF1R inhibitors alone. Based on this, Zhang et al. [65] explored the influencing factors of the TME through the SCS analysis of samples from different tissue types collected from treatment-naive patients, including 17 CRC liver metastasis, 18 CRC, and 16 hepatocellular carcinoma patients. By comparing the metastatic foci with CD45+ cells from different tissues, the researchers classified the immune cells in the metastatic foci into three categories: malignancy-associated, niche-associated, and combo-associated. Among them, Tex (exhaustion CD8+ T cells) subpopulations shared the same TCR between primary and metastatic lesions, indicating that Tex may have common non-exhaustible precursor cells in the peripheral region. Zhang suggested that the identified subpopulation of myeloid cells of M type, SPP1+ macrophages, may play a role in promoting tumor metastasis. In another study, Gao et al. [60] collected 97 samples from 24 patients with a pathological diagnosis of CRC liver metastasis, and conducted single-cell transcriptome sequencing, space transcriptome sequencing, and multiple immunofluorescence techniques, which revealed dynamic changes in immune cells in CRC liver metastasis. Gao et al. [66] found the presence of immunosuppressive cells in liver metastases, Observing a sharp upward trend in SPP1+ macrophages and MRC1+CCL18+M2-like macrophages. Among them, MRC1+CCL18+M2-like macrophages had expression characteristics similar to Kupffer cells, indicating that they might be derived from Kupffer cells in liver tissues. The macrophages in the metastases attained an inhibitory state, confirming the potential inhibitory effect of the TME in CRC liver metastasis patients. Similarly, Pelka et al. [67] developed a systematic method to identify cell types, basic programs, and cell communities based on SCS, resulting in the determination of a periodic spatial cellular interaction network coordinating multicellular immune responses in mismatch repair-deficient and mismatch repair-proficient CRC.

In addition, Zhang et al. [14] conducted scRNA-Seq on CRC liver metastatic cancer tissues and adjacent liver tissues and identified 12 cell subsets across six cell types, including tumor epithelial cells, T cells, myeloid cells, endothelial cells, fibroblasts, and B cells. The expression of 445 cell cluster deregulated genes was clustered into six groups, and functional enrichment was performed to analyze the related pathways in tumor epithelial cells and T cell populations. Simultaneously, de Vries et al. [68] evaluated the expression of 36 immune cell markers at the single-cell level of 35 CRC tissues, 26 tumor-related lymph nodes, 17 normal colorectal mucosa, and peripheral blood samples from 19 CRC patients. The results revealed a novel group of congenital lymphocytes, which expressed the T cell surface antigen Leu-9 (CD7+), neural cell adhesion molecule 1 (CD56+), and protein tyrosine phosphatase receptor type C (CD45RO+) at high levels, while did not express IL-7 receptor (CD127−) (Lin-CD7+CD127−CD56+CD45RO+), which was enriched in CRC tissues and showed cytotoxicity. The subset of cells expressed the integrin subunit αE (CD103+) and leucocyte surface antigen Leu-23 (CD69+) (CD103+CD69+) phenotypes specifically and was most abundant in immunogenic mismatch repair deficient CRC. The findings suggest that both innate and adaptive immune cell populations are present within tumors, suggesting that the anti-tumor characteristics of tumors can be used for multi-target combined therapies.

## 4. Conclusions

**Figure 1:**
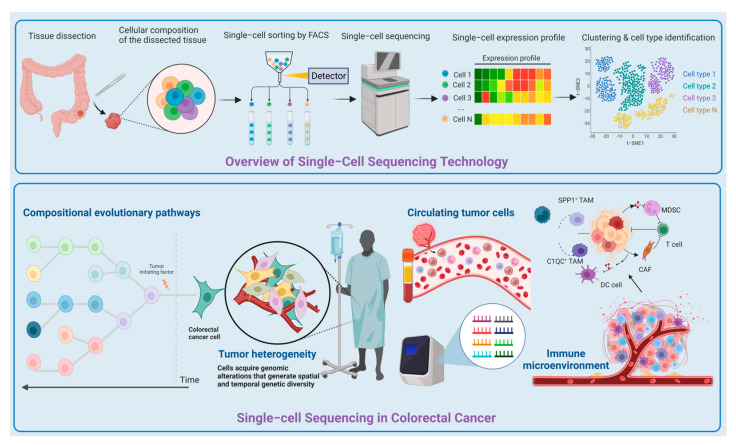
Schematic diagram of single-cell sequencing and its application in colorectal cancer.

CRC is the third most common cancer after breast and lung cancers. With the improvement of single-cell technology and the reduction of cost, the high gene coverage and reliability and stability of results obtained with this method have enabled researchers to systematically and comprehensively analyze the physiological and pathological functions of specific cell subsets. The schematic diagram of the entire manuscript is shown in Figure 1. In the field of research on CRC, SCS can further reveal the cloning and differentiation of cells, cell heterogeneity in tissues, cell types involved in CRC, changes in immune cells, the gene expression regulation network, dynamic changes between transcription and protein abundance, and the operating mechanism of molecular networks or cell networks in complex tissues. In addition, it has also shown great potential in the determination of T cell and macrophage distribution, understanding antigenicity in tumor heterogeneity, and studying the transformation of clone diversity and drug resistance in invasion and immunotherapy. The findings will pave the way for research on the prevention and treatment of CRC at the single-cell level. More importantly, the integration of single-cell and multi-genome sequencing technology can provide us with better means to understand the molecular network and interaction mechanism between cells. Although the results of SCS cannot be verified in cellular experiments at present, and the isolation and identification of specific cell subsets is still an expensive and challenging process, the further development of SCS can help understand the occurrence and development of CRC and the key factors of diagnosis and treatment more directly, which can help conduct clinical transformation research more actively.

## Figures and Tables

**Table 1:** Summary of current single-cell RNA-sequencing methods.

SCS	Sample Type	Species	Year	cDNA Coverage	Amplification Technology	Technical Introduction	Advantage	Insufficient	Platform Used
Tang RNA-Seq [27]	Oocyte or blastomere	Mouse	2009	Full-length with 3′-biased	PCR after polyA tailing	cDNA is synthesized using poly T base as a primer, and poly base A is added at the 3′ end as the poly T base-binding site of the second chain	The full length of the transcript can be measured, and gene expression detection is more sensitive and accurate	Strong bias to the 3′ end, less cell flux, and more expensive	Sequencing by Oligo Ligation Detection (SOLiD), Applied Biosystems, Foster, CA, USA
Smart-Seq [6]	Mammalian cells	Human and mouse	2013	Full-length with 3′-biased	Template switching-based PCR	RNA is hybridized with primers containing oligo (dT). Following this, several template-free C nucleotides are added to generate the first chain, and the poly (C) suspension is only added to the full-length transcript. Then, an oligonucleotide primer is hybridized with poly (C) to synthesize the second chain	The method has good sequence coverage. It can facilitate the detection of selective transcript isomers and SNV. It improves the coverage at the mRNA 5′ end, which is a technology for detecting full-length mRNA	It is a non-chain-specific amplification method, and the length of transcripts is biased. The method cannot be used to efficiently transcribe sequences larger than 4 kb. The disadvantages are a preferentially increasing abundance of transcripts and material loss during purification	Illumina’s HiSeq 2000, GAIIx or MiSeq instruments, Illumina, San Diego, CA, USA
Smart-seq2 [28]	N/A	N/A	2014	Nearly full-length	Template switching-based PCR	Two to five template-free C-nucleotides are added to the 3′ end of the cDNA. Then, a template-converting oligonucleotide (TSO) is added to produce a lock nucleotide modification at the 3′ end. Limited cyclic amplification of cDNA is observed after the first strand reaction	No purification is required, transcript coverage is better, and the production and levels of localizable sequences are enhanced	Non-chain-specific amplification, and only sequencing of poly (A)+RNA, less cell flux, more expensive	HiSeq 2000, 2500 or MiSeq instrument, Illumina, San Diego, CA, USA
CEL-Seq [29]	blastomeres	C. elegans	2014	3′ tag (UTR)	in vitro transcription	It is a sequencing method based on linear amplification. Sequences are amplified by in vitro transcription (IVT). By connecting the T7 promoter to oligodT primers, IVT can be initiated after cDNA synthesis	The contamination among samples is reduced considerably. The read length preference is very low, and the chain is specific	The error rate is relatively low, but both amplification and PCR have sequence preference, strong 3′ preference, high-abundance transcripts are preferentially amplified, and at least 400 pg of total RNA is required	Illumina HiSeq2000, Illumina, San Diego, CA, USA
Quartz-seq [30]	N/A	N/A	2013	Full-length with 3′-biased	PCR after polyA tailing	The strategy for inhibition PCR is used to make primers that self-hybridize to form a pan structure to reduce by-products, and small fragments of the second-strand cDNA form a hairpin structure	Greatly reduces PCR by-products, uses an efficient enzyme to adapt to single-tube reaction, optimize the conditions for inversion and second-strand cDNA synthesis, and reduces contamination by small fragments	Amplification bias is easily introduced owing to the difference in GC content	Illumina HiSeq 1000/2000, Illumina, San Diego, CA, USA
STRT-seq [31]	Oocytes	Mouse	2014	5′ tag (TSS)	Template switching-based PCR	The combination of molecular markers and microfluidic technology is used to quantitatively estimate the initial mRNA expression	High cell flux, relatively cheap	Only the transcript end is detected, and the sensitivity of gene expression detection is low, which is not suitable for the analysis of alternative splicing and allele expression, among others	SOLiD system, Thermo Fisher Scientific, Waltham, MA, USA
Drop-seq [32]	Retinal cells	Mouse	2015	3′ tag (UTR)	Template switching-based PCR	Micro drop-based approach. Each cDNA is labeled with a cell-specific barcode and UMI	Possibility of low-cost, rapid library preparation, single-cell high-throughput analysis, and multigroup analysis	A microfluidic platform is required, and the sensitivity of single-cell gene is low	Illumina NextSeq 500, Illumina, San Diego, CA, USA

## Data Availability

Not applicable.
